# Age-Related Olfactory Dysfunction: Epidemiology, Pathophysiology, and Clinical Management

**DOI:** 10.3389/fnagi.2020.00208

**Published:** 2020-07-07

**Authors:** Kenji Kondo, Shu Kikuta, Rumi Ueha, Keigo Suzukawa, Tatsuya Yamasoba

**Affiliations:** Department of Otolaryngology—Head and Neck Surgery, Graduate School of Medicine and Faculty of Medicine, The University of Tokyo, Tokyo, Japan

**Keywords:** aging, olfactory receptor neurons, basal cells, regeneration, olfactory bulb, olfactory cortex, neurodegenerative diseases

## Abstract

Like other sensory systems, olfactory function deteriorates with age. Epidemiological studies have revealed that the incidence of olfactory dysfunction increases at the age of 60 and older and males are more affected than females. Moreover, smoking, heavy alcohol use, sinonasal diseases, and Down’s syndrome are associated with an increased incidence of olfactory dysfunction. Although the pathophysiology of olfactory dysfunction in humans remains largely unknown, studies in laboratory animals have demonstrated that both the peripheral and central olfactory nervous systems are affected by aging. Aged olfactory neuroepithelium in the nasal cavity shows the loss of mature olfactory neurons, replacement of olfactory neuroepithelium by respiratory epithelium, and a decrease in basal cell proliferation both in the normal state and after injury. In the central olfactory pathway, a decrease in the turnover of interneurons in the olfactory bulb (OB) and reduced activity in the olfactory cortex under olfactory stimulation is observed. Recently, the association between olfactory impairment and neurodegenerative diseases, such as Alzheimer’s disease (AD) and Parkinson’s disease (PD), has gained attention. Evidence-based pharmacotherapy to suppress or improve age-related olfactory dysfunction has not yet been established, but preliminary results suggest that olfactory training using odorants may be useful to improve some aspects of age-related olfactory impairment.

## Introduction

Olfaction is a sense that allows the detection of odors in the surrounding environment. Olfaction is necessary to identify food, predators, and sexual partners for most of the wildlife, being indispensable for species survival. Moreover, in humans, olfaction not only guarantees greater safety by allowing the detection of fire, gas leakage, and spoiled foods but also a greater quality of life through the appreciation of food, wine, and pleasant smells. Nonetheless, olfactory impairment has traditionally received less attention compared to visual and auditory impairment. Thus, it may even be difficult for healthy individuals to understand the inconvenience of olfactory impairment. However, patients with olfactory impairment face a series of daily-life problems (Miwa et al., 2001; Brämerson et al., 2007; Gopinath et al., 2012a; Croy et al., 2014) as well as psychological issues including depression, anxiety, and other negative emotions (Croy et al., 2014).

Several pathoetiologies, including chronic rhinosinusitis, viral infection, head trauma, and intake of toxic drugs are associated with the development of olfactory dysfunction (Hummel et al., 2017). Along with these pathologies, age is one of the most important factors associated with human olfactory dysfunction (Schiffman, 1997; Brämerson et al., 2007; Doty and Kamath, 2014; Mobley et al., 2014; Attems et al., 2015). As other sensory functions such as hearing and vision, olfactory ability deteriorates with aging, and the majority of the patients with the complaint of olfactory impairment are middle-aged and elderly patients. In line with the rapid growth of the geriatric population in developed countries, the number of individuals with olfactory impairment is also expected to rapidly grow. For elderly people with decreased physical and social activity, food may be a source of joy in their daily life (Markovic et al., 2007), consequently, olfactory dysfunction may lead to impairing their quality of life. Recent studies have also demonstrated that patients with age-related neurodegenerative diseases, such as Alzheimer’s disease (AD) and Parkinson’s disease (PD), develop olfactory dysfunction from the early stages of the diseases (Kovács, 2004; Barresi et al., 2012; Doty, 2017). More recent studies in the general population have also demonstrated that olfactory dysfunction is an independent risk factor for mortality (Gopinath et al., 2012b; Pinto et al., 2014; Devanand et al., 2015; Schubert et al., 2017; Liu et al., 2019), suggesting that olfaction may serve as a biomarker for systemic life activity. Effective day-to-day management of olfactory dysfunction as well as the development of new treatment strategies for age-related olfactory dysfunction are therefore important goals to support healthy, successful aging.

While there has been rapid progress in understanding the molecular mechanisms mediating olfaction (Ihara et al., 2013; Takeuchi and Sakano, 2014), the pathogenetic processes underlying age-related human olfactory dysfunction, and, in particular, the changes in the olfactory neural system, remain largely unknown. This review article, therefore, aimed to shed light on age-related changes occurring in the olfactory neural system during olfactory deterioration, both within the context of the normal aging process and under pathological conditions.

## Overview of the Anatomy of Olfactory Neural Pathways

The human olfactory mucosa is located in the superior part of the olfactory cleft, overlying the cribriform plate as well as the superior part of the nasal septum and the middle and superior turbinates. In many animal species, including mice, rats, and dogs, the olfactory mucosa covers a large proportion of the nasal cavity (Mery et al., 1994; Kavoi et al., 2010). In contrast, in humans, the relative surface of olfactory mucosa is very small, covering only a few cm^2^ in each nostril (Holbrook et al., 2011).

**Figure 1:**
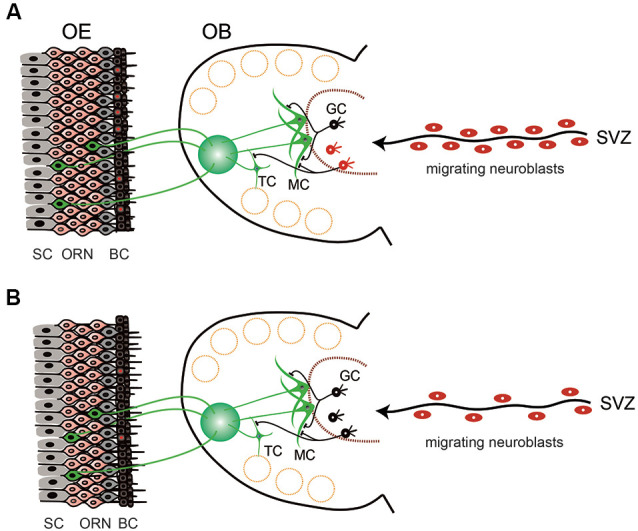
Schematic view of the structure and cell dynamics of the olfactory neuroepithelium (OE) and olfactory bulb (OB) in young **(A)** and aged **(B)** mice. **(A)** OE is composed of layers of supporting cells (SC), olfactory receptor neurons (ORN), and basal cells (BC). BCs continuously undergo cell division to give rise to new neuronal cells (BCs with red nuclei). ORNs extend axons to the glomerulus of the OB, where they make synaptic connections with the neurites of mitral cells (MC) and tufted cells (TC). There is also continuous cell generation in the stem cell population in the subventricular zone (SVZ). Generated neuroblasts then migrate to reach the OB and differentiate into the interneurons [mostly into granule cells (GC)] and contribute to the modulation of olfactory information. **(B)** In aged animals, the cell division in the BCs is decreased and the number of ORNs is also reduced. Cell division in the SVZ is reduced, as well as the number of neuroblasts migrating to the OB. However, the number of granule cells in the OB is almost stable, suggesting that the turnover of granule cells is reduced and the lifespan of the cells is prolonged (black GCs).

The olfactory mucosa is composed of the neuroepithelium and underlying lamina propria. The neuroepithelium is a pseudostratified epithelium, with the basal cells residing at the bottom of the epithelium above the basement membrane (Figure 1A). There are two distinct types of basal cells, horizontal basal cells (HBCs), and globose basal cells (GBCs). Above the basal cells is a layer of neural cells with various differentiation status, ranging from the immature (basal) to the mature (apical) status (Figure 1A). Olfactory receptor neurons (ORNs) are bipolar neurons, whose dendrites extend to the surface of the epithelium and axons extend to the olfactory bulb (OB). The end of ORN dendrites forms a knob-like structure, known as the olfactory vesicle, from which several olfactory cilia emanate. The most apical zone of the olfactory neuroepithelium is occupied by the nuclei and/or cytoplasm of the supporting cells (Moran et al., 1982; Morrison and Costanzo, 1990; Jafek et al., 2002). The lamina propria beneath the basement membrane contains vessels, olfactory nerve bundles, and Bowman’s glands. The function of Bowman’s glands remains unclear, but it is speculated that they contribute to: (1) the protection of olfactory cilia; (2) the transport of odorants; (3) the prevention of mucosal infection through the secretion of antimicrobial proteins; and (4) the biochemical detoxification of chemicals through biotransformation enzymes (Getchell and Getchell, 1991; Mellert et al., 1992; Matarazzo et al., 2002; Ling et al., 2004).

Because olfaction is a chemical sensor that detects evaporated chemicals, olfactory receptors need to be exposed to the external environment. The olfactory mucosa is part of the airway mucosa. External exposure is a distinct feature of the olfactory sensory system from the visual and auditory systems, which perceive physical stimuli made of light and sound, respectively, have their receptors protected inside the body. The peripheral olfactory organ is therefore always at risk of being injured by extrinsic pathogens and chemicals. Conversely, olfaction plays an indispensable role in survival, contributing to food detection, predator avoidance, and mating in the wildlife. To meet these diverse needs, the mammalian olfactory neural system has a unique regenerative capacity compared to other sensory systems. The most distinct feature of such regenerative capacity is the continuous proliferation of basal cells in the neuroepithelium. GBCs are a type of neural stem cells, which continuously undergo cell division even in undamaged conditions and give rise to new ORNs (Figure 1A). When the neuroepithelium is injured, such proliferative activity is upregulated so the neuroepithelium is regenerated rapidly (Matulionis, 1975; Graziadei and Graziadei, 1979; Hurtt et al., 1988; Schwob et al., 1992, 1995; Genter et al., 1995; Bergman et al., 2002; Ducray et al., 2002; Schwob, 2002; Suzukawa et al., 2011). Although it is unknown how long it takes for the human olfactory neuroepithelium to recover from damage, in rats and mice the olfactory neuroepithelium morphologically recovers from experimentally-induced mucosal injury within one month (Graziadei and Graziadei, 1979; Schwob, 2002; Suzukawa et al., 2011). In undamaged conditions, HBCs are quiescent cells, but in the event of severe mucosal damage, HBCs function as stem cells and proliferate to give rise to each cell type in the neuroepithelium (Farbman, 1990; Schwob et al., 1992, 2017; Schwob, 2002; Beites et al., 2005; Brann and Firestein, 2014). This regenerative ability is retained until old age, though its efficacy decreases (Morrison and Costanzo, 1995; Hahn et al., 2005; Suzukawa et al., 2011; Brann and Firestein, 2014).

**Figure 2:**
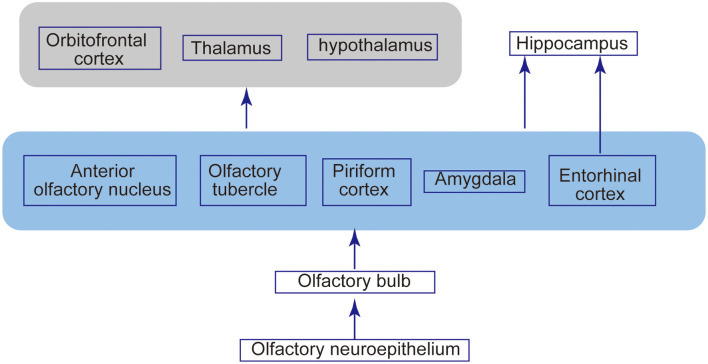
Olfactory structures and neural connections. Odor molecules are perceived by odorant receptors expressed on the olfactory receptor neurons in the neuroepithelium. Olfactory signals are then sent intracranially to the OB and relayed to the second-order neurons, mitral cells, and tufted cells. These second-order neurons extend their axons along the lateral olfactory tract toward the structure of the primary olfactory cortex, such as the anterior olfactory nucleus, piriform cortex, and entorhinal cortex. This olfactory cortex has a projection extending to other brain areas, including the thalamus, hypothalamus, orbitofrontal cortex, and hippocampus.

Olfactory signals from ORNs relay to second-order neurons, namely the mitral cells and tufted cells in the OB. ORNs and mitral/tufted cells make synapses, forming signal-processing modules named glomerulus (Figure 1A). In mice, each ORN expresses only one of more than 1,000 types of olfactory receptors. The axons of ORNs expressing the same olfactory receptors converge on only a few glomeruli. Functional studies have demonstrated that an odorant receptor can be activated by multiple odorant molecules, whereas an odorant molecule can activate multiple odorant receptors. Therefore, an odorant is identified as a unique combination of olfactory receptor responses, which eventually leads to a unique activation profile of olfactory glomeruli in the OB. This mechanism is thought to enable the olfactory system to discriminate thousands of odors (Ressler et al., 1994; Mori et al., 1999). These second-order neurons extend their axons along the lateral olfactory tract toward the structure of the primary olfactory cortex, such as the anterior olfactory nucleus, piriform cortex, and entorhinal cortex. Odor processing may also involve other central brain areas, including the hippocampus, amygdala, and orbitofrontal cortex (Gottfried, 2010; Figure 2).

Although the mitral cells and tufted cells are generated only during the embryonic period, there is a continuous turnover of interneurons in the OB, as in the neuroepithelium. Continuous cell generation occurs in the stem cell population located in the subventricular zone (SVZ). The generated neuroblasts migrate to the OB, where they differentiate into interneurons (mostly into granule cells; Whitman and Greer, 2009; Lazarini and Lledo, 2011; Mobley et al., 2014) and contribute to the reorganization of the olfactory neural pathways (Figure 1A).

## Age-Related Changes in Human and Animal Olfactory Function in Physiological Conditions

Many studies have revealed that the human olfactory function, including the ability to detect, differentiate, and identify odors, declines with age. Doty et al. (1984) investigated the olfactory identification ability in a variety of age generations using the Pennsylvania smell identification test and found that significant olfactory deterioration starts from the age in 60 in males and 70 in females, with females maintaining a superior ability in each generation. Hummel et al. (2007) examined more than 3,000 normal subjects using Sniffin’ Sticks, a battery of olfactory tests, and reported that the sum of threshold, discrimination, and identification scores (TDI scores) declined with age, with odor thresholds declining most dramatically compared to odor discrimination and odor identification. In Japan, Saito et al. (2006) demonstrated using the Japanese olfactory identification test (OSIT-J) that olfactory identification ability declined from the age of 50 onward.

Konstantinidis et al. (2006) reported that the identification of unpleasant odors is independent of age, suggesting that the decline in olfactory sensitivity may depend on the type of odorants. Conversely, Sinding et al. (2014) reported that age-related loss in olfactory sensitivity is similar for light and heavy molecules. It was also reported that the detection thresholds for lavandin oil (a highly complex substance) and n-butanol (a single compound) are elevated to a similar extent in subjects over 70 years compared to controls under 30 (Stevens and Spencer, 1994).

In laboratory animal studies, behavioral tests have also demonstrated that several aspects of olfactory function decline with age. For example, olfactory perceptual learning, an ongoing process whereby animals learn to discriminate odorants, is impaired with aging (Moreno et al., 2014). Additionally, older mice require more training sessions and make more errors than younger mice for olfactory discrimination and have significantly higher detection thresholds for ethyl acetate vapor (Patel and Larson, 2009). Psychophysical experiments in rats found the highest olfactory sensitivity to occur at 13 months of age and the lowest sensitivity at 25 months of age and older. However, aged rats did not show any age-related deterioration in learning ability in an olfactory discrimination task compared to young adults (Kraemer and Apfelbach, 2004). Older rats were less reactive than younger rats in a test of cat odor avoidance, but they expressed similar amounts of cat odor-induced Fos (a neuronal activity marker) in the posterior accessory OB, a critical region for processing predator odor stimulus. Thus, the loss of reactivity may be due to changes in the more central olfactory processing (Hunt et al., 2011).

## Prevalence of Age-Related Olfactory Dysfunction in Humans

Several epidemiological surveys have examined the prevalence of olfactory dysfunction in the general population. The methods included questionnaire-based self-assessment of olfactory function (Hoffman et al., 1998; Lee et al., 2013; Rawal et al., 2016) and use of psychophysical olfactory tests (Larsson et al., 2000; Murphy et al., 2002; Bramerson et al., 2004; Landis et al., 2004; Ross et al., 2008; Vennemann et al., 2008; Shu et al., 2009; Wehling et al., 2011; Gopinath et al., 2012b; Mullol et al., 2012). In general, the prevalence of olfactory impairment based on self-assessment is lower than that based on olfactory tests. For example, Hoffman et al. (1998) estimated based on a questionnaire survey from 42,000 households in the United States that 1.4% of American adults experience olfactory problems. Lee et al. (2013) reported based on a national survey in the Korean population (4,000 households) that the prevalence of subjective olfactory dysfunction in adults was 4.5%. In contrast, in a population-based study in Sweden, the olfactory function of 1,387 adults was tested using a smell identification test and 19.1% of respondents showed olfactory dysfunction (Bramerson et al., 2004). Likewise, in the population-based survey in Germany (*n* = 1,312, 25–75 years) using a smell identification test, the prevalence of olfactory dysfunction was estimated as 21.6% (Vennemann et al., 2008). This discrepancy is probably due to common unawareness of olfactory deterioration, especially in the elderly population (Murphy et al., 2002; Shu et al., 2009; Wehling et al., 2011). Furthermore, the prevalence of olfactory dysfunction based on olfactory tests varies among studies, possibly because of differences in the population examined, type of olfactory test, and definition of olfactory impairment. Despite such discrepancies in their methods, these studies have consistently demonstrated that the prevalence of olfactory dysfunction increases with age. For example, the National survey by Hoffman et al. (1998) in the United States demonstrated that the prevalence of subjective olfactory dysfunction was 2.0% in the 55–64 age range but was 4.6% in the 75 years and older population. Furthermore, in a population-based survey using an odor identification test in the United States, the prevalence of olfactory impairment was 6.1% in individuals aged 53–59 years but was 29.2% in individuals aged 70–79 years, and as much as 62.5% in the group aged 80–97 years (Murphy et al., 2002).

Some studies have estimated the risk of developing olfactory dysfunction by testing human subjects after specific time intervals and the results of these studies have demonstrated that risk increases with age. For example, the risk of developing olfactory dysfunction during the following 5 years was 4.1% in the 53–59 age category but 21% in the 70–79 age category and 47.1% in the 80–97 age category (Schubert et al., 2011). In another study that evaluated 57–85-year-old American adults twice at 5-year intervals, the olfactory identification ability deteriorated more rapidly in older individuals and men than in their respective counterparts (Pinto et al., 2015).

## Risk Factors for Age-Related Olfactory Dysfunction

Several risk factors have been identified for olfactory dysfunction. While most of the studies assessed olfactory dysfunction in general, a few have focused on identifying risk factors specific to olfactory dysfunction in the aging process. The results, however, vary depending on the studies.

Most of the epidemiological studies have demonstrated that males have a higher risk of being hyposmic, that is, have a reduced ability to smell and detect odors (Doty et al., 1984; Vennemann et al., 2008; Schubert et al., 2012, 2015; Dong et al., 2017). Beyond gender, ethnicity may influence olfactory decline. Dong et al. (2017) reported that the incidence of anosmia (loss of the sense of smell) in the older population is higher in black people compared to white people. Pinto et al. (2015) also reported that olfaction in African Americans deteriorates more rapidly than in Whites. Moreover, adults with Down’s Syndrome (DS) show significantly poorer odor thresholds and odor identification abilities than age- and cognitively-matched control subjects (Murphy and Jinich, 1996; Nijjar and Murphy, 2002). Also, adults with DS show poorer odor identification ability than children and younger adults with DS, suggesting that age-related olfactory dysfunction progresses more rapidly in DS than in normal subjects (Nijjar and Murphy, 2002).

Another possible risk factor for olfactory dysfunction is smoking. In cross-sectional population-based surveys, ongoing chronic smoking increased the risk for impairment of olfactory function (Vennemann et al., 2008; Schubert et al., 2012; Glennon et al., 2019). A systematic review and meta-analysis also demonstrated that current smoking, but not former smoking, was associated with a significantly increased risk of olfactory dysfunction, suggesting that the effects of smoking on olfaction may be reversible (Ajmani et al., 2017). However, olfactory impairment in smokers has been reported to persist 15 years after quitting (Siegel et al., 2019). In animal studies, intranasal administration of tobacco solution induced degeneration of the olfactory neuroepithelium (Ueha et al., 2016a,b, 2018c).

Other suggested risk factors include heavy alcohol use (Schubert et al., 2011; Rawal et al., 2016; Glennon et al., 2019), sinonasal diseases (Schubert et al., 2011, 2012; Rawal et al., 2016), history of head injury (Rawal et al., 2016), income <110% poverty threshold (Rawal et al., 2016), and body weight loss (Gopinath et al., 2012b; Aiello et al., 2019). On the other hand, regular exercise was reported to be associated with a lower 10-year cumulative incidence of olfactory impairment (Schubert et al., 2013).

To determine the prognostic factors of olfactory dysfunction, London et al. (2008) assessed olfactory function in 542 patients using olfactory test scores on two occasions, which were separated from one another by a varying duration ranging from 3 months to 24 years. Patient age, the severity of initial olfactory loss, and the patient-reported duration of dysfunction at the first testing were significant predictors of improvement in olfactory function. Etiology, sex, the time between the two tests, and initial smoking behavior were not significant predictors. Moreover, Doty et al. (2011) measured odor identification ability in a population-based cohort of 1,222 twins and singletons of very old age. Sex, age, cognitive function, and smoking were significant predictors of olfactory test scores. The study also demonstrated that the effects of heritability on odor identification decline with age, suggesting that adverse environmental factors contribute more than genetic factors to such olfactory deterioration, especially at older ages (Doty et al., 2011).

## Pathology of the Age-Related Olfactory Mucosa in Humans and Laboratory Animals

Because of the difficulty to obtain samples, information regarding age-related histological changes in the human olfactory mucosa is limited. Biopsy and cadaver studies have demonstrated that the surface of the olfactory mucosa decreases with age. Moreover, within the olfactory mucosa area, disruption of the zonal distribution of supporting, ORNs, and basal cells and patchy replacement of olfactory neuroepithelium by respiratory epithelium could be observed with increasing age (Nakashima et al., 1984; Paik et al., 1992; Holbrook et al., 2011).

In rodents, it has been shown that the olfactory mucosa surface decreases with age, especially in the anterior portion of the nasal cavity, and that the olfactory mucosa undergoes degenerative changes such as the irregular boundary of olfactory and respiratory regions, reduced number of ORNs, and inclusion bodies, as also seen in humans (Loo et al., 1996; Breckenridge et al., 1997; Rosli et al., 1999; Kondo et al., 2009). The lesions in the neuroepithelium and underlying Bowman’s glands tend to be spatially co-localized, suggesting a close association between their pathogenesis (Kondo et al., 2009).

Age-related changes in organs may be due to both physiological degenerations associated with increasing lifetime and age-related changes associated with pathologies. It is difficult to discriminate these two causes for the change in the clinical setting. In laboratory animals, even animals kept under very clean air conditions show degenerative changes in the olfactory neuroepithelium (Loo et al., 1996). Therefore, age-related mucosal degenerative changes could be, at least in part, a result of mere physiological aging. Conversely, in the clinical setting, the incidence of postviral olfactory disorders is more frequent among middle-aged and elderly patients, and recovery becomes increasingly incomplete with increasing age (Reden et al., 2006), suggesting that aging may affect the susceptibility of the olfactory neural system against damaging factors and its regenerative capacity after injury. In laboratory animals, rats can still detect food odor with more than 90% of the olfactory mucosa being degenerated (Youngentob et al., 1997), suggesting taht the peripheral olfactory system has a large spare ability. Should this also be the case in humans, patients who have just noticed olfactory deterioration may be in the final stages of olfactory neuroepithelial degeneration after the long-term latent progress of degeneration.

## Age-Related Decrease in Basal Cell Proliferation

Because the olfactory neuroepithelium has self-renewal capacity, the balance between olfactory neurogenesis and cell death is responsible for the maintenance of an adequate number of ORNs. Basal cell proliferation both in undamaged tissues and after injury decreases with age (Fung et al., 1997; Weiler and Farbman, 1997; Ducray et al., 2002; Kondo et al., 2010; Jia and Hegg, 2015; Ueha et al., 2018b; Figure 1B). Suzukawa et al. (2011) evaluated age-related changes in neuroepithelial regeneration after chemical injury in mice and demonstrated that: (1) the chronological pattern in neuronal cell proliferation and differentiation was similar among the different age groups; (2) the extent of neuroepithelial cell proliferation after injury decreased with age; and (3) the final histological recovery of the olfactory neuroepithelium and the innervation of the OB was significantly lower in the aged group than in younger age groups. These results suggest that the age-related decline in olfactory neuroepithelial regeneration capacity is associated with a decreased proliferative activity, rather than with changes in the neuronal differentiation process.

While apoptosis, the other cellular event that may be involved in the maintenance of the olfactory neuroepithelium cell population, has been investigated in several studies, it remains controversial to date whether or not apoptosis increases with age (Fung et al., 1997; Robinson et al., 2002; Conley et al., 2003; Kondo et al., 2010; Ueha et al., 2018b).

## The Molecular Basis Underlying Age-Related Neuroepithelial Changes

The molecular basis underlying age-related changes in the olfactory neuroepithelium remains largely unclear. While there have been an increasing number of studies addressing the molecular mechanisms underlying olfactory neural regeneration, most of these did not specifically investigate the aging process.

During basal cell proliferation, several transcription factors operate concomitantly to further or stop the cell division process (Beites et al., 2005; Schwob et al., 2017). The expression of cyclin D, a transcription factor promoting cell proliferation, decreases with age (Legrier et al., 2001). The expression of various growth factors such as epidermal growth factor, insulin-like growth factor-1 (IGF-1), and neuropeptide Y signaling decreases with age (Enwere et al., 2004; Chaker et al., 2015; Jia and Hegg, 2015; Ueha et al., 2018b), which may also be associated with the decrease in basal cell proliferation.

The aging of stem cells, in addition to extrinsic factors, may also be associated with the age-related decrease in basal cell proliferation. For example, the number of basal cells which express the neural stem cell marker Musashi-1decreases with age (Watanabe et al., 2007). Telomerase-deficient mice, in which there is a shortening of telomere lengths, show more partial neuroepithelial recovery compared with wild type mice after olfactory mucosal injury (Watabe-Rudolph et al., 2011). Child et al. (2018) have demonstrated that in transgenic mice in which the diphtheria toxin is expressed in ORNs under the control of the olfactory marker protein (OMP) promoter, ORNs apoptosis increases and cell turnover in the neuroepithelium is accelerated. In this model, the neurogenetic capacity of basal cells is “exhausted” and neuroepithelial degeneration persists even after toxin expression has ceased. This suggests that when excess basal cell division occurs due to the repeated damage of ORNs, the capacity of the basal cell to proliferate is eventually lost and neuroepithelial degeneration occurs (Child et al., 2018).

In the aging process, cells are exposed to many sources of oxidative stress and can stop mitotic activity irreversibly. Some affected cells undergo apoptosis, but others survive and begin to secret inflammatory cytokines such as interleukin-6 (IL-6) and tumor necrosis factor-alpha (TNF-α). This phenomenon is designated as the senescence-associated secretory phenotype (Zhu et al., 2014). A similar process may occur in the olfactory mucosa, as supported by the higher concentration of IL-6 in the olfactory mucosa of aged mice compared to young mice (Ueha et al., 2018b). In a human study, hyposmia was correlated with increased IL-6 concentrations in serum and nasal mucus (Henkin et al., 2013). It has also been shown that in a transgenic mouse model of olfactory inflammation, in which TNF-α expression is induced specifically within the olfactory epithelium, inflammation induces the suppression of basal cell proliferation with the resulting degeneration of the olfactory neuroepithelium (Lane et al., 2010). These observations suggest that the elevation of inflammatory cytokines in the aged olfactory neuroepithelium may be associated with the loss of ORNs and suppression of basal cell proliferation.

DNA microarray analysis of olfactory mucosa in senescence-accelerated mouse (SAM) has demonstrated that changes in the expression of genes associated with chemosensory detection, immune barrier function, xenobiotic metabolism, cell cycle progression, and cell death were particularly prominent in old SAM strains (Getchell et al., 2003, 2004). Conversely, Rimbault et al. (2009) have demonstrated using microarray and quantitative polymerase chain reaction (PCR) that, when gene expression in the olfactory mucosa was compared between newborn, 9-week, and 22-month-old Brown Norway rats, overall gene expression did not change considerably across ages, with only 0.25% of the transcripts showing differential expression. This suggests that age-related changes in gene expression in the olfactory mucosa may be strain-dependent.

## Age-Related Changes in the Expression of Odorant Receptors

Several studies have explored whether the expression pattern of odorant receptors (OR) changes with age. Khan et al. (2013) have reported using Nanostring assay that the gene expression profile of odorant receptors in the C57B6 mice strain is almost stable within the 2–31 month age range. Rimbault et al. (2009) have also demonstrated using microarray and quantitative PCR that the OR gene expression is not different between 9-week and 22-month-old Brown Norway rats. Conversely, in another mouse study conducted by Ueha et al. (2018b) using microarray analysis, the expression of many OR genes changed with age. Also, in mice, although the overall number of ORNs decreases with age, the extent of the loss of ORNs differs according to the type of odorant receptor the neurons express (Lee et al., 2009). In contrast, the number of ORs expressed on individual ORNs is stable (Lee et al., 2009). In humans, the response of individual ORNs to odors is not affected by aging, while the specificity of the response of each ORN to the odor decreases with age (Rawson et al., 2012). If the expression level of specific ORs on the olfactory mucosa decreases with age, elderly people may show decreased sensitivity for these specific odors. In other words, it is possible that the quality of the odorant perception when smelling changes with age, because the combination of ORs activated by the odor could change. Parosmia is a dysfunction in the field of smell detection characterized by the inability to properly identifying an odor’s natural smell. In humans, parosmia is often observed in cases of postviral or traumatic olfactory disorders, whereas it is rarely observed in age-related slowly progressing olfactory decline (Nordin et al., 2007). This finding suggests that in humans, although the overall number of ORNs decreases with age, the proportion of each OR gene expression in the olfactory mucosa may not be different between young and older individuals.

## Age-Related Changes in the Structure and Cell Dynamics of the Olfactory Bulb

Human studies have reported that the volume of the OB, the glomerular layer thickness, the number of glomeruli, and the concentration of mitral cells per unit area all decrease with increasing age (Bhatnagar et al., 1987; Meisami et al., 1998; Yousem et al., 1998). Studies in rodents have demonstrated more mixed results. In mice, the bulb volume did not change (Richard et al., 2010), or rather increased with age (Mirich et al., 2002). The number of mitral cells is not altered considerably during physiological aging (Richard et al., 2010). In rats, from 24 to 30 months of age, a significant decrease occurs in the volume of the bulb layers, and the number of mitral cells decreases (Hinds and McNelly, 1977). The number of olfactory axodendritic synapses in the glomeruli and the total volume of glomerular dendrites, especially in the glomerulus layer, both decrease with age (Richard et al., 2010). Such decreases appear to reflect the decrease in synapse formation between the dendrites of the neurons, as well as the decrease in the number of ORNs (Buschhüter et al., 2008). It is thus suggested that the atrophic changes in the OB may be in part secondary to changes in the ORNs. The number of synapses in the glomeruli appears to decline less markedly with age than the number of ORNs, and a significant increase in the number of synapses per ORN occurs in the oldest group studied (33 months), suggesting a compensatory increase in the relative number of synapses per ORN (Hinds and McNelly, 1979, 1981).

The number of stem cells and their proliferation in the SVZ, and the migration of new neurons as well as their integration into the neural system in the OB, are all reduced with age, and the elimination of adult-born neurons in the OB is promoted with age (Figure 1B; Maslov et al., 2004; Honda et al., 2009; Choi et al., 2010; Bouab et al., 2011). Because the survival of granule cells is influenced by odorant stimulation and food-intake activity (Yokoyama et al., 2011), the decrease in the migration of new neurons may be a secondary change due to the reduced sensory input caused by the decrease in the number of ORNs (Yoshihara et al., 2012).

Interestingly, the granule cell density in the OB does not significantly decrease with age (Richard et al., 2010), but rather may increase (Enwere et al., 2004). This suggests that the turnover in the granule cell population decreases, with granule cells living longer in aged animals than in young animals (Sui et al., 2012). The relevance of these findings to olfactory function remains unclear, but the decrease in the turnover of granule cells may lead to a less flexible reorganization of neural connections in response to a new odorant. It has also been suggested that neurogenesis, rather than the total number of interneurons, is important for fine olfactory discrimination (Enwere et al., 2004).

If the change in the cell turnover of the OB represents the primary change, changes in cell turnover may reflect changes in the availability of growth factors. It has been reported that the expression of IGF-1 decreases with age (Ferrari et al., 2003; Chaker et al., 2015) and that insulin binding decreases in the OB with age, probably because of the decrease in the insulin receptor number in the OB (Tchilian et al., 1990). The accumulation of oxidative stress and the resulting cell aging may also cause age-related changes in the OB (Vaishnav et al., 2007; Romero-Grimaldi et al., 2008).

## Age-Related Changes in the Central Olfactory Pathway and Olfactory Cortex

To date, there is limited evidence regarding age-related changes in the central olfactory pathway and olfactory cortex regions such as the anterior olfactory nucleus, olfactory tubercle, piriform cortex, amygdala cortical nucleus, and entorhinal cortex. However, changes in the central olfactory pathway also appear to be involved in age-related functional deficits.

Human morphometrical studies using magnetic resonance imaging (MRI) have yielded mixed results. One study suggests that among the olfactory-related brain structures, the volume of the piriform cortex and that of the amygdala cortex do not reduce with age, while the volume of the orbitofrontal cortex significantly decreases (Shen et al., 2013). Another study examining normal subjects across ages showed that the volume of the OB and tract showed an initial increase up to the 4th decade of life, followed by a decrease with increasing age (Yousem et al., 1998). Conversely, functional MRI studies suggest that activation of the central olfactory region, including the piriform cortex, entorhinal cortex, and amygdala, and of the orbitofrontal cortex decreases in older age (Cerf-Ducastel and Murphy, 2003; Wang et al., 2005). During early aging, the activity of the primary olfactory cortex under olfactory stimulation is not correlated with age, while the activity of the secondary and higher central olfactory structures (prefrontal cortex, insular cortex, and orbitofrontal cortex) is negatively correlated. This suggests that in the early aging process, an age-related functional decline in the human brain is more prominent in the secondary and higher-order central olfactory structures than in other regions (Wang et al., 2017). Furthermore, a positron emission tomography (PET) study has demonstrated that age-related reduction in the binding potential for the striatal dopamine transporter in the putamen is associated with the age-related olfactory deficit (Larsson et al., 2009). Consistent with these findings, the olfactory event-related potential shows an age-related decrease in its amplitude and processing speed with increasing age (Yousem et al., 1999; Murphy et al., 2000; Suzuki et al., 2001).

In animal studies, the histological analysis of rat brains has also demonstrated that the volume of the piriform cortex does not decline with age (Curcio et al., 1985). Conversely, electrophysiological recordings in the anterior piriform cortex have demonstrated that the synaptic α-amino-3-hydroxy-5-methyl-4-isoxazolepropionic acid (AMPA) receptor is decreased during aging, suggesting that glutamatergic synaptic function changes with age (Gocel and Larson, 2013).

## Putative Gene Polymorphism Associated With Age-Related Olfactory Dysfunction in Humans

Recent analyses have demonstrated the association of gene variants with olfactory dysfunction, but few of these analyses have addressed age-related olfactory dysfunction. Dong et al. (2015) performed the first genome-wide meta-analysis on the sense of smell among 6,252 U.S. older adults of European descent. The results suggest that the microtubule-associated protein tau locus may play a role in regulating the sense of smell in older adults. Furthermore, the effect of the brain-derived neurotrophic factor (BDNF) val66met polymorphism on olfactory function changes was examined in a large-scale, longitudinal population-based sample (Hedner et al., 2010). The magnitude of the olfactory decline in the older age cohort was larger for the VAL homozygote carriers than for the MET carriers.

## Association of Age-Related Changes in Trigeminal and Autonomic Nervous Systems With Olfactory Dysfunction

Another sensory system in the nasal cavity is the trigeminal sensory system. It has been suggested that the trigeminal system contributes to olfaction. Compared to younger subjects, older people have a reduced sensitivity to the intranasal trigeminal system (Frasnelli and Hummel, 2003). Furthermore, patients with olfactory dysfunction have lower scores in the lateralization task than controls, indicating decreased trigeminal sensitivity compared to healthy controls (Hummel et al., 2003). Since it has been reported that the loss of trigeminal sensitivity reduces olfactory sensitivity (Husner et al., 2006), the function of the trigeminal and olfactory nervous system may be linked. There is a neural connection between these two nervous systems in the OB (Finger and Böttger, 1993; Schaefer et al., 2002). Both the trigeminal and autonomic nervous system innervations in the olfactory mucosa show age-related changes (Chen et al., 1993). The distribution of adrenergic innervation in the human olfactory mucosa reveals a decrease in the innervation density of blood vessels over 60 years of age. These age-related changes may be involved in age-related olfactory sensitivity.

## Olfactory Dysfunction Associated With Neurodegenerative Diseases

More and more attention has been paid to the association between olfactory dysfunction and neurodegenerative diseases whose incidence increases with aging (Kovács, 2004; Barresi et al., 2012). The olfactory central pathway has recently gained more attention clinically, since the early pathological changes of neurodegenerative diseases, including AD and PD, occur in the central olfactory pathways.

**Figure 3:**
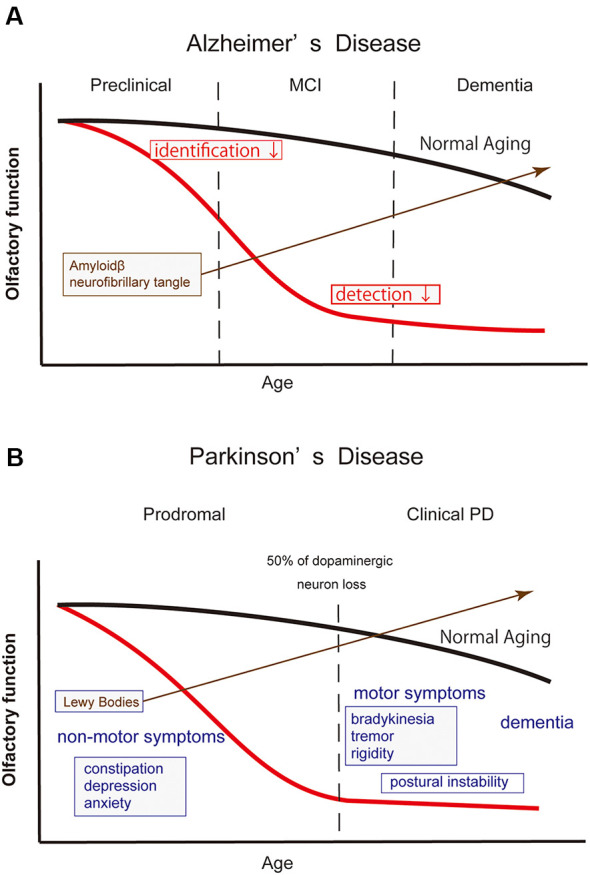
Schematic drawing illustrating the olfactory dysfunction and time course of Alzheimer’s disease (AD) and Parkinson’s disease (PD). In both neurodegenerative diseases, the emergence of olfactory dysfunction precedes their definite diagnosis. The red and black lines in the drawing indicate the time course of olfactory function in patients and normal elderly individuals, respectively. **(A)** In AD, olfactory dysfunction emerges in its preclinical phase when cognitive function is still preserved. At the time, however, the accumulation of amyloid β already begins in the brain structures, including in the olfactory pathway. Odor identification ability deteriorates first, followed by a worsening in odor detection. **(B)** Definite diagnosis of PD is made by the emergence of motor symptoms when approximately 50% of dopaminergic neurons are lost in the substantia nigra. Olfactory dysfunction emerges in the prodromal phase, along with other non-motor symptoms. Lewy bodies are already observed in the olfactory system, including the OB. In clinical PD, the severity of olfactory dysfunction does not appear to be correlated with the disease duration or disease stages, suggesting that the degenerative changes in the olfactory neural structures have progressed at the time of diagnosis.

In AD, the most frequent neurodegenerative disease globally, characteristic pathological changes such as senile plaque and neurofibrile changes appear in the OB, anterior olfactory nucleus, and entorhinal cortex (Braak and Braak, 1991; Hyman et al., 1991; Kovács et al., 1999; Daulatzai, 2015). The OB as well as the olfactory neuroepithelium show degenerative changes in patients with AD (Trojanowski et al., 1991; Yamagishi et al., 1998; Kovács et al., 2001; Attems et al., 2005; Thomann et al., 2009). Patients with AD show olfactory dysfunction at the early phase of the disease (Doty et al., 1987; Serby et al., 1991; Peters et al., 2003; Hori et al., 2015; Silva et al., 2018; Jung et al., 2019; Figure 3). Odor identification deteriorates first followed by odor detection (Murphy et al., 1990; Serby et al., 1991). When elderly individuals with normal cognitive function were prospectively followed up, subjects with olfactory dysfunction showed a faster cognitive decline (Dintica et al., 2019) and developed mild cognitive impairment (MCI) more frequently than the subjects with normal olfaction (Wilson et al., 2007; MacDonald et al., 2018). Furthermore, MCI patients with olfactory dysfunction transited to AD more frequently than patients with normal olfaction (Devanand et al., 2000; Roberts et al., 2016). Therefore, olfactory dysfunction is expected to be one of the biomarkers to predict progression from normal cognitive status or MCI to AD (Adams et al., 2018; Windon et al., 2020). One study reported that a specific pattern of olfactory identification deficit may differentiate AD from age-related olfactory loss (Woodward et al., 2018).

PD represents the second-largest neurodegenerative population after AD. Patients with PD show olfactory dysfunction (Doty et al., 1988; Iijima et al., 2008; Watanabe et al., 2017), and the olfactory test score is not correlated with motor function, disease duration, or disease stages (Doty et al., 1988; Iijima et al., 2008; Figure 3). The Movement Disorders Society has officially adopted olfactory dysfunction as a supporting diagnostic criterion for clinical PD (Postuma et al., 2015), as well as a supporting research criterion for prodromal PD (Berg et al., 2015). Hyposmia has a high discriminatory power to differentiate PD from differentials such as multiple system atrophy, progressive supranuclear palsy, drug-induced parkinsonism, and essential tremor, with >80% sensitivity and specificity (Mahlknecht et al., 2016). Older people with olfactory dysfunction are at higher risk of developing PD (Ross et al., 2008; Berg et al., 2012; Chen et al., 2017; Fullard et al., 2017). Two recent studies have reported that the relative risk of developing incident PD in hyposimc subjects over non-hyposmic subjects up to a 10-year follow-up period was 3–4 (Chen et al., 2017; Mahlknecht et al., 2020). These findings suggest that there is a long prodromal phase of the illness and that the patients in this phase may be underdiagnosed as merely having “age-related olfactory dysfunction.”

Characteristic pathological changes of PD such as Lewy bodies and the accumulation of alpha-synuclein are observed at the early phase of the disease (Braak et al., 2004; Funabe et al., 2013). Histological studies have suggested that the deposition of Lewy bodies appears preferentially in the olfactory tract, including the OB and anterior olfactory nucleus (Hubbard et al., 2007; Funabe et al., 2013). Interestingly, the presence of Lewy bodies in the brain is associated with olfactory dysfunction in otherwise asymptomatic elderly individuals (Ross et al., 2006; Wilson et al., 2011) and this is thought to represent a presymptomatic stage of PD. Taken together, olfactory dysfunction could be a useful screening biomarker to identify those at high risk for developing PD. It has also been reported that olfactory impairment predicts cognitive decline in early PD patients (Baba et al., 2012; Fullard et al., 2016; Domellöf et al., 2017).

Early diagnosis and intervention of patients at risk and earlier stages of the disease appear to be essential for any successful neuroprotection. In PD, the degenerative change in the dopaminergic neurons in the substantia nigra has already progressed severely (approximately 50% of loss) when neurologists can make a diagnosis according to the accepted clinical diagnostic criteria (Becker et al., 2002). Neuroprotective therapy starting at such an advanced stage of the disease may not be effective enough to stop the degenerative process. Therefore, the identification of non-motor symptoms, especially the olfactory function, may be useful for the early diagnosis and treatment of PD. However, as a single marker, hyposmia is not specific to predict PD, and therefore additional markers are needed for an accurate diagnosis. Berg et al. (2015) have proposed a formula to estimate the probability of prodromal PD using several parameters. Jennings et al. (2017) also have proposed a two-step approach (olfactory test followed by dopamine transporter imaging) to identify individuals from the general population at risk for conversion to a clinical diagnosis of PD.

## Management Strategies for Age-Related Olfactory Dysfunction

The prevention of olfaction dysfunction may lead to happier and more successful aging. In the case of olfactory impairment, clinical management may help patients to overcome the difficulties associated with their impairment. Although, several drugs have been tested for the treatment of age-related sensorineural olfactory dysfunction including zinc, vitamins, and herbal medicines, no evidence-based medicine has been established to improve age-related olfactory dysfunction (Miwa et al., 2019).

Recently, olfactory training has been reported to be useful for the treatment of sensorineural olfactory disorders (Hummel et al., 2009; Damm et al., 2014). The original method reported by Hummel et al. (2009) required patients to expose themselves twice daily to four odors [phenyl ethyl alcohol (PEA): rose, eucalyptol: eucalyptus, citronellal: lemon, and eugenol: cloves]. Olfactory training has been reported to improve age-related olfactory loss (Birte-Antina et al., 2018), although further studies are warranted to confirm the efficacy.

The mechanism underlying odor stimulation-dependent improvement is unclear, but as described above, the number of interneurons in the OB is regulated depending on odor stimulation (Yokoyama et al., 2011). Also, in the olfactory neuroepithelium, odor stimulation is important to maintain the survival of newly-generated ORNs, especially in the critical periods of making synapses (Kikuta et al., 2015).

Although still at the experimental stage, the intranasal application of growth factors, gene therapy, and stem cell transplantation have been tested as a treatment of sensorineural olfactory degeneration (Choi and Goldstein, 2018; Kurtenbach et al., 2019). Intranasal application of drugs/genes/cells may also be useful for age-related olfactory dysfunction because the most prominent pathological feature of the age-related changes in olfactory neuroepithelium is the reduced basal cell proliferation (Weiler and Farbman, 1997; Kondo et al., 2010; Suzukawa et al., 2011). The olfactory mucosa is exposed to an airway and offers the advantage of easy accessibility. Animal studies have demonstrated that intranasal application of fibroblast growth factor-2 and IGF-1 promote neuroepithelial regeneration after chemical injury in aged mice (Fukuda et al., 2018). Another study in which IGF-1 was administered subcutaneously to aged mice demonstrated that while low-dose IGF-1 administration increases the numbers of olfactory progenitors, immature ORNs, and mature ORNs in the olfactory neuroepithelium (OE), high-dose IGF-1 administration increases only the number of immature ORNs, with a concurrent increase in apoptotic cells (Ueha et al., 2018a). This finding suggests that in designing drug therapies, the use of an appropriate dose is important.

Intranasal administration of drugs has also been extensively studied as a treatment of central nervous system diseases, because the olfactory mucosa may be used as a route to deliver drugs to the intracranial space bypassing the blood-brain barrier (Chapman et al., 2013). The provision of daily-life advice, especially to guarantee patient safety and the appreciation of food is also important to manage age-related olfactory impairment. With olfactory deterioration, patients tend to fail the detection of hazardous odors, such as gas leakage and fire smoke odors. In a family of an elderly couple, possibly none can detect such hazardous odors. For such patients, the use of odor detection machines is recommended (Miwa et al., 2001). Patients may also fail to notice the smell of spoiled food. In such a situation, it is recommended to pay attention to food conditions by checking the expiration date label, especially in the summertime.

Another problem to be addressed is malnutrition due to olfactory impairment. It is reported that the addition of flavor to the food may increase appetite and improve the nutritional condition (Schiffman and Warwick, 1993). Conversely, patients with a neural disorder such as postviral and traumatic olfactory dysfunction, frequently experience parosmia, which causes food such as fish, oily food, some vegetables and fruits, and fermented goods to have unpleasant odors during the recovery period. Therefore, adequate food choices while cooking are important to maintain the joy of the meals.

## Author Contributions

All authors wrote the manuscript. SK and KK designed the figures. All authors reviewed and approved the final version of the manuscript.

## Conflict of Interest

The authors declare that the research was conducted in the absence of any commercial or financial relationships that could be construed as a potential conflict of interest.
